# Global trends in medical education accreditation

**DOI:** 10.1186/s12960-021-00588-x

**Published:** 2021-05-20

**Authors:** Deborah Bedoll, Marta van Zanten, Danette McKinley

**Affiliations:** Foundation for Advancement of International Medical Education and Research (FAIMER), Philadelphia, USA

**Keywords:** Accreditation standards, Medical education, Health professions education, Regulation

## Abstract

**Background:**

Accreditation systems in medical education aim to assure various stakeholders that graduates are ready to further their training or begin practice. The purpose of this paper is to explore the current state of medical education accreditation around the world and describe the incidence and variability of these accreditation agencies worldwide. This paper explores trends in agency age, organization, and scope according to both World Bank region and income group.

**Methods:**

To find information on accreditation agencies, we searched multiple online accreditation and quality assurance databases as well as the University of Michigan Online Library and the Google search engine. All included agencies were recorded on a spreadsheet along with date of formation or first accreditation activity, name changes, scope, level of government independence, accessibility and type of accreditation standards, and status of WFME recognition. Comparisons by country region and income classification were made based on the World Bank’s lists for fiscal year 2021.

**Results:**

As of August 2020, there were 3,323 operating medical schools located in 186 countries or territories listed in the *World Directory of Medical Schools.* Ninety-two (49%) of these countries currently have access to undergraduate accreditation that uses medical-specific standards. Sixty-four percent (*n* = 38) of high-income countries have medical-specific accreditation available to their medical schools, compared to only 20% (*n* = 6) of low-income countries. The majority of World Bank regions experienced the greatest increase in medical education accreditation agency establishment since the year 2000.

**Conclusions:**

Most smaller countries in Europe, South America, and the Pacific only have access to general undergraduate accreditation, and many countries in Africa have no accreditation available. In countries where medical education accreditation exists, the scope and organization of the agencies varies considerably. Regional cooperation and international agencies seem to be a growing trend. The data described in our study can serve as an important resource for further investigations on the effectiveness of accreditation activities worldwide. Our research also highlights regions and countries that may need focused accreditation development support.

## Background

There are currently over 3000 medical schools providing education and training to aspiring physicians around the world. The medical education curriculums, experiences offered, available resources, length of study, etc., vary widely depending on regional, political and other contextual factors. This variability in educational models, combined with the rapid increases in the number of medical schools worldwide [1] and increasing international mobility for education and employment [2] necessitate oversight of quality assurance, such as formal accreditation systems, to ensure medical educational institutions function appropriately [3]. For the purpose of this paper, we use the definition of accreditation as described by van Zanten et al. [4], “a process by which a designated authority reviews and evaluates an educational institution using a set of clearly defined criteria and procedures”.

Accreditation systems in medical education aim to assure various stakeholders, including students, educators in postgraduate educational programs, employers, and patients, that graduates are ready to further their training or begin practice. Oversight of the educational content and pedagogical methods is necessary to ensure that the learning needs of the students are met and endeavor to ultimately impact the quality of medical care provided to patients. While there should also be significant consequences for educational institutions that do not meet the standards, an important aim of the accreditation process should be encouraging ongoing institutional improvement and fostering the dissemination of best practices, both regionally and globally.

The development and sustainability of educational quality assurance systems is supported by various international organizations worldwide. The World Health Assembly in its Global Strategy on Human Resources for Health: Workforce 2030, encouraged all countries to have accreditation for medical and other health training programs by 2020 [5]. The World Medical Association also supports quality assurance mechanisms to promote trust in the health workforce [6]. The World Federation for Medical Education (WFME) Recognition Programme aims to provide an independent, transparent and rigorous method of ensuring that accreditation of medical schools worldwide is at an internationally accepted and high standard [7]. As part of the Recognition Programme, WFME evaluates compliance of accrediting agencies with pre-defined criteria [8].

Since 2005, the Foundation for Advancement of International Medical Education and Research (FAIMER®) has been gathering and publishing data on accreditation activities worldwide. Their Directory of Organizations that Recognize/Accredit medical schools (DORA) lists organizations that recognize, authorize, or certify medical schools and/or medical education programs and related data [9]. Summary data from DORA of accreditation activities around the world showed that while over half of all countries with medical schools indicate that there is a national process of accrediting medical education programs, there was considerable variation in scope of authority and level of enforcement [4]. For example, accreditation is managed and implemented by various organizations/agencies around the world, including professional bodies or associations, such as associations of medical schools, statutory bodies such as Medical Councils, or by national accreditation authorities that conduct quality assurance reviews of all higher education, including health professions education [10]. While the creation of a separate medical education accreditation system, in addition to an accreditation system already in place to review an entire university (including the medical school) could be viewed as redundant, authorities that compared health-care discipline specific accreditation systems with general higher education accreditation processes have argued for the importance of specific quality assurance focused on health professions such as medicine [11, 12].

The purpose of this paper is to explore the current state of medical education accreditation around the world and describe the incidence and variability of these accreditation agencies worldwide. By tracking the founding years of accreditation organizations and comparing our data against that found in 2008 [4], we show the growth and change of such academic accreditors over time, as well as updating the data available for future research. This descriptive study explores trends in agency organization and scope according to both World Bank region and income group, and highlights regions that may need focused accreditation development support.

## Methods

### Search strategy

**Fig. 1:**
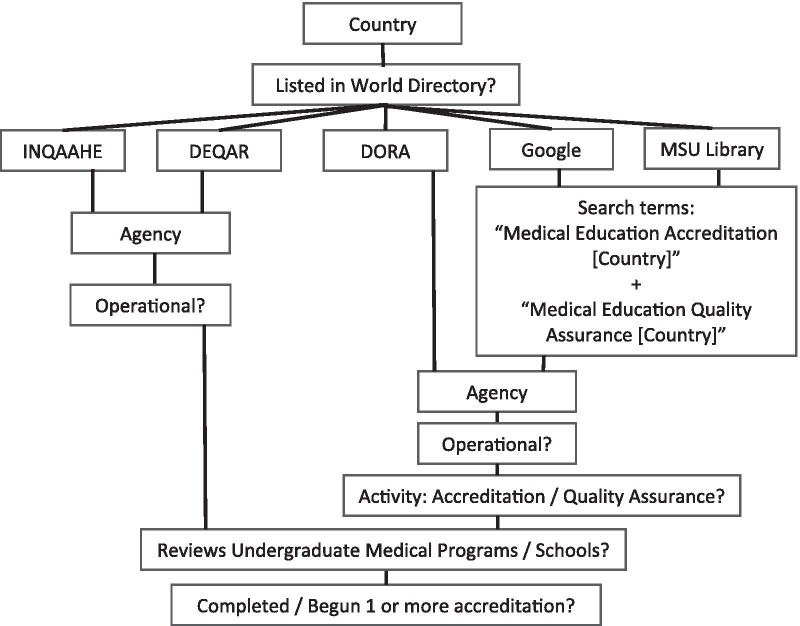
A visual representation of our search strategy. Countries and agencies that did not meet the criteria listed as questions below were not included in the analysis

To find information on accreditation agencies in each country and to identify trends in organization and scope, in August 2020 we searched DORA [9], the International Network for Quality Assurance Agencies in Higher Education (INQAAHE) [13], a worldwide association of organizations that are active in quality assurance in higher education, and the Database of External Quality Assurance Results (DEQAR) [14], a database of reports and decisions on higher education institutions and programs from agencies registered in the European Quality Assurance Register (EQAR). We supplemented our search with the Google search engine and the University of Michigan Online Library, using the terms “medical education accreditation ‘[Country]’” and “medical education quality assurance ‘[Country]’” to find further information about agency histories and relationships or additional accreditation agencies that were not listed in the above databases. Figure 1 below shows a visual representation of our search strategy.

As of August 2020, there were 3,323 operating medical schools located in 186 countries or territories listed in the *World Directory of Medical Schools* (*World Directory*) [15]. Countries and territories that did not have at least one operational medical school listed on the *World Directory* by August 1, 2020, were not included in the analysis. A complete list of accreditation agencies included in this analysis can be found in Appendix 1.

### Analysis

Each identified accreditation agency was screened to identify its operational status. Agencies that were non-operational were excluded from the analysis. To be included, an accreditation agency must have the term “accreditation” or “quality assurance” as an activity, apply this activity to undergraduate programs or schools that include basic medical education programs, and have demonstrably begun or completed at least one accreditation. Agencies performing consultative visits only were not included.

All included agencies were recorded on a spreadsheet along with their date of formation or first accreditation activity. If date of first medical school/program accreditation was 5 years or more later than date of formation, the later accreditation date was used. Where it could be shown that an agency had undergone name changes, the date of first accreditation of their parent agency was used. Information recorded for each agency included national or international scope, whether accreditation standards were accessible, if the agency is currently WFME recognized or had applied for WFME recognition as of September 1, 2020, and supplemental links and details. In addition, to provide contextual background information, the level of independence from the national government was investigated. Government relationship was recorded as “public” if the agency was originally formed and still managed as a government agency, “independent” if the agency was formed by the government but managed autonomously, and “private” if the agency was not formed by an act of government.

To be included in this review, agency standards documents were available in English language, or in a document that could be translated into English using Google Translate (https://translate.google.com/). To be recorded as offering medical-specific accreditation in this review, an agency’s standards were available online or provided through email. If an agency could be verified as providing some type of accreditation, but their standards could not be located or successfully translated, the agency was recorded as having general accreditation standards.

To classify agencies as having medical-specific or general accreditation standards, we referred to the WFME Standards for Basic Medical Education, 2015 [12]. These standards comprise basic curricular standards including biomedical sciences (B 2.3), medical ethics (B 2.4.3), medical research methods (B 2.2.2), evidence-based medicine (B 2.2.3), and patient contact (B 2.5.2), must ensure adequate clinical training facilities (B 6.2.2), and must specify the amount of time spent in training in major clinical disciplines (B 2.5.4). We selected these seven requirements as being unique to the health professions, and representative of health-professions-specific standards. For the purpose of this review, the terms “health-professions” and “medical” are used interchangeably. Agency standards were reviewed and classified as medical-specific if they included two or more requirements that focused on content comparable to the above standards. Agencies with standards that included one or no requirements related to the seven Basic Medical Education standards above were classified as offering general accreditation. Agencies with standards that did not stipulate medical education requirements beyond the inclusion of a health-professions expert in the accreditation team were recorded as having general accreditation standards.

We used counts and percentages to describe the number of agencies by accreditation type, location, founding date, and level of government independence. Country region and income classifications were based on the World Bank’s lists for fiscal year 2021 [16]. Comparisons by country region and income classification were made. We compared these results to the findings of van Zanten et al. [4] to identify trends.

## Results

Table 1 presents information on the level of accreditation that is available for undergraduate medical programs or schools in countries with at least one known medical school (*n* = 186) by World Bank region. Ninety-two (49%) of these countries currently have access to undergraduate accreditation that uses medical-specific standards. This accreditation is provided by 71 accreditation agencies, of which 23 (32%) are currently recognized by WFME.

**Table 1:** Countries with access to accreditation for undergraduate medical programs or schools by World Bank Region

Accreditation type	World Bank Region							
	Eastern Asia and Pacific (*n* = 23)	Europe and Central Asia (*n* = 48)	Latin America and the Caribbean (*n* = 37)	Middle East and North Africa (*n* = 20)	North America (*n* = 2) North America (*n* = 2)	South Asia (*n* = 7)	Sub-Saharan Africa (*n* = 42)	Not listed by World Bank (*n* = 7)
Medical-specific	12 (52%)	18 (38%)	26 (70%)	12 (60%)	2 (100%)	4 (57%)	13 (31%)	5 (71%)
General	9 (39%)	30 (62%)	8 (22%)	6 (30%)	0	3 (42%)	7 (17%)	0
None or data unknown	2 (9%)	0	3 (8%)	2 (10%)	0	0	22 (52%)	2 (29%)

There is wide variability in the availability of medical-focused accreditation across the regions, ranging from 31% (*n* = 13 countries) in Sub-Saharan Africa to 100% (*n* = 2 countries) in North America. Of all types of accreditation agencies for which we were able to determine their government relationship (*n* = 189 agencies), about half (*n* = 94) of the organizations are public, 35% (*n* = 67) are independent, and 15% (*n* = 28) are private. Of the medical-education-specific accreditation agencies, 42% (*n* = 30) are public, 37% (*n* = 26) are independent, and 21% (*n* = 15) are private.

Table 2 presents information on the level of accreditation available for undergraduate medical programs or schools in countries with medical schools (*n* = 186 countries) by World Bank economic group. Sixty-four percent (*n* = 38) of high-income countries have medical-specific accreditation available to their medical schools, compared to only 20% (*n* = 6) of low-income countries. More than half of low-income countries did not have undergraduate accreditation systems that we could discern.

**Table 2:** Countries with access to accreditation for undergraduate medical programs or schools by World Bank Economic Group

Economic group	Medical accreditation	General accreditation	None or unknown
Low-income countries (*n* = 30)	6 (20%)	8 (27%)	16 (53%)
Medium-income countries (*n* = 90)	43 (48%)	34 (38%)	13 (14%)
High-income countries (*n* = 59)	38 (64%)	21 (36%)	0
Not listed by World Bank (*n* = 7)	5 (71%)	0	2 (29%)

**Fig. 2:**
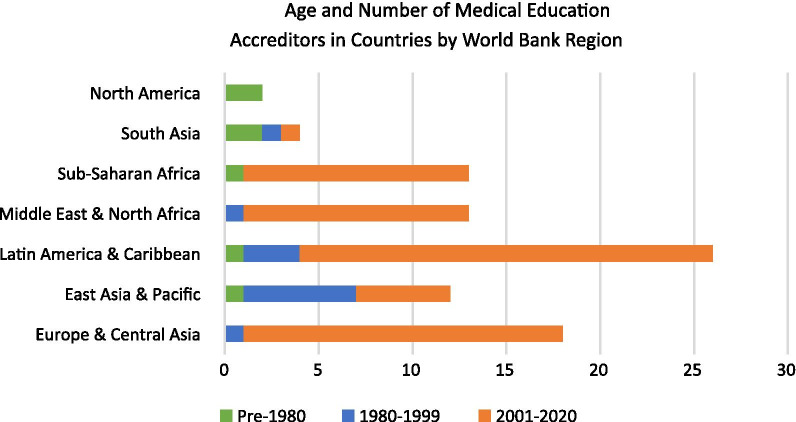
Medical education accreditation agencies by World Bank Region

To examine scope and tenure of the agencies, we examined trends by region. Findings are summarized in Fig. 2, which contrasts the number of medical education accreditation agencies created in each time period and highlights the recent acceleration of agency creation. Four of the nine regions experienced the greatest increase in medical education accreditation agency establishment since the year 2000; however, East Asia and the Pacific saw the greatest growth in the years 1980–1999, while both South Asia and North America developed most of their agencies pre-1980. The Latin America region has the greatest number of medical education accreditation agencies, followed by the Europe & Central Asia region.

**Fig. 3:**
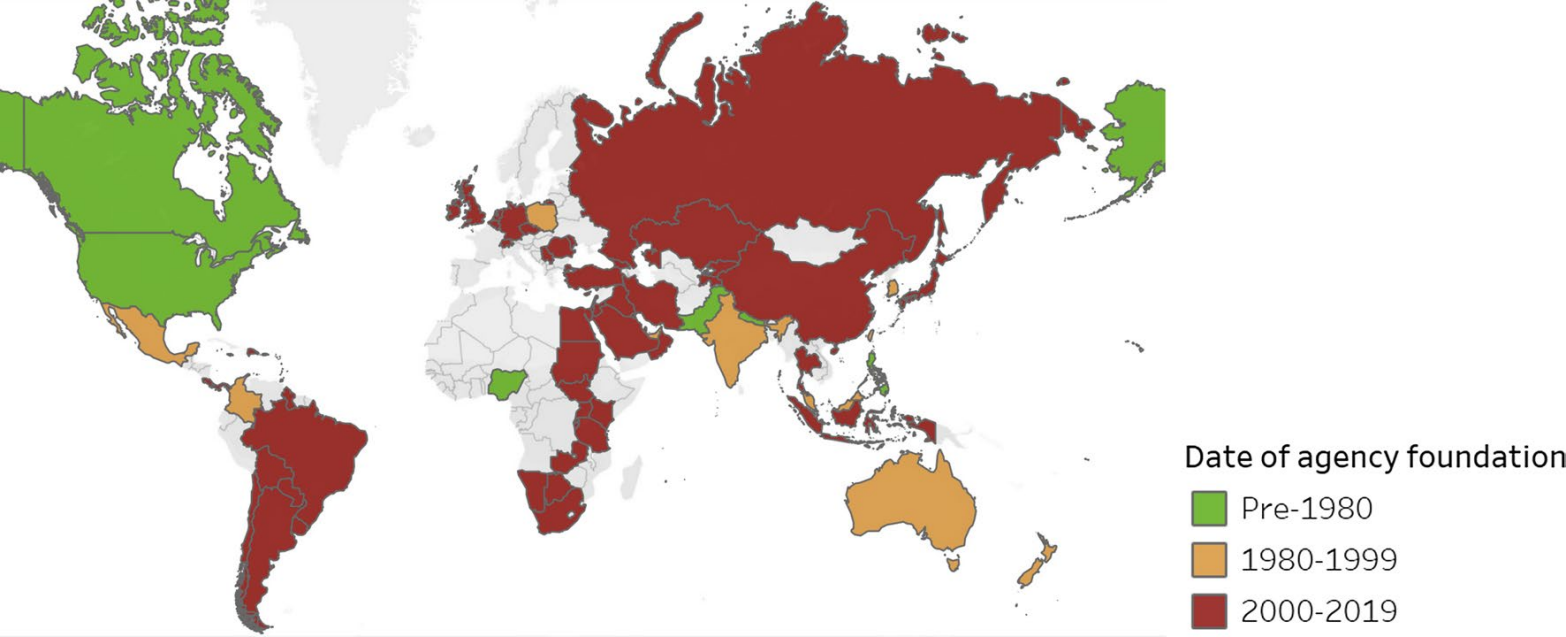
Countries with undergraduate medical education accreditation agencies

Figure 3 shows the age of undergraduate medical education accreditors on a global map. Medical education accreditation is now available in most larger countries, although it exists in only about half of countries with medical schools. Areas that are still under-represented in this type of accreditation include western Africa, southern and eastern Europe, northern South America, and Scandinavia.

**Fig. 4:**
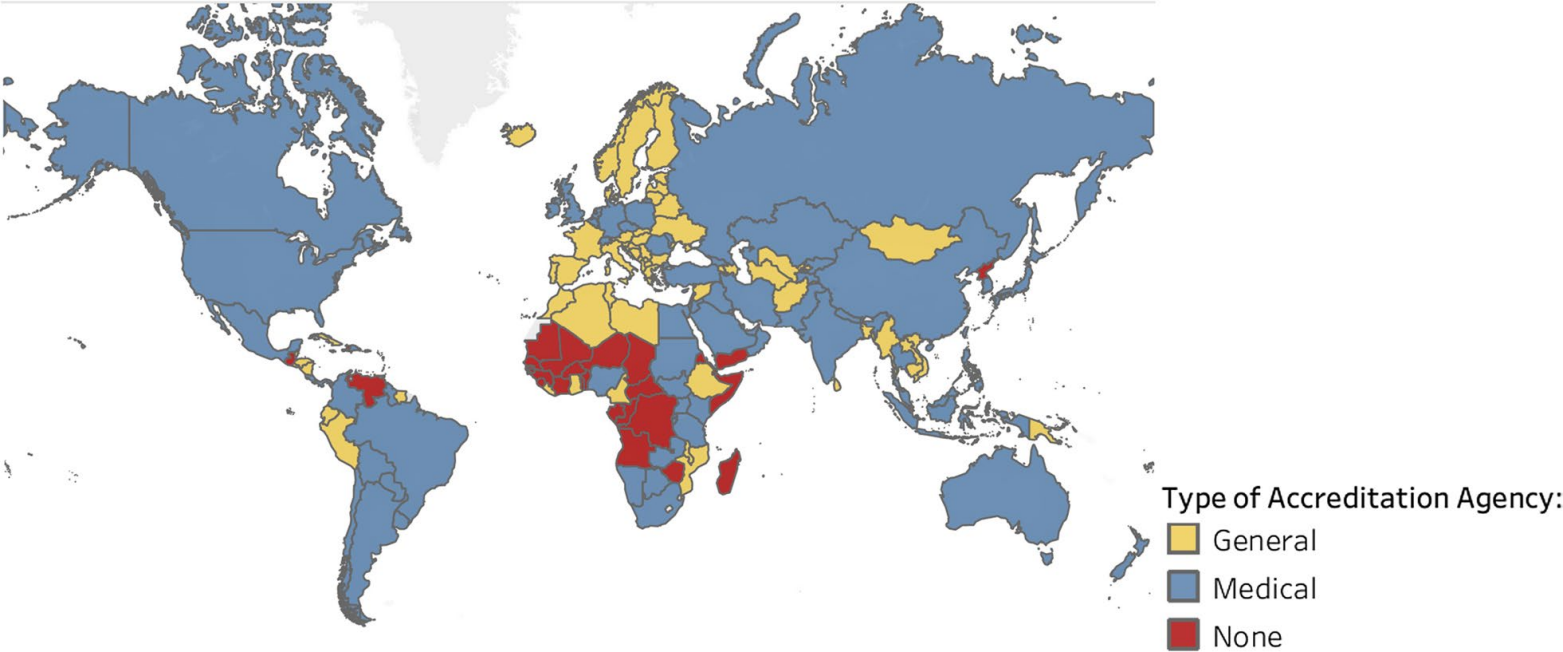
Countries with accreditation agencies that review undergraduate medical programs or schools

Figure 4 shows the type of undergraduate accreditation agency in each country on a global map. Most smaller countries in Europe, South America, and the Pacific only have access to general accreditation, and many countries in Africa do not have medical accreditation available. Detailed information on the agencies scope and trends over time are reported in the next section of the paper.

### Sub-Saharan Africa

Of the 42 countries in the World Bank’s Sub-Saharan Africa region that have medical schools listed in the *World Directory,* 20 (48%) have known accreditation authorities. Although this is the lowest percentage of countries with academic accreditation of all World Bank regions, this number has increased almost 300% since van Zanten’s 2008 review [4], in which only seven countries had accreditation authorities. The 20 countries are served by a total of 28 different organizations, half of which (*n* = 14) provide medical-education accreditation, while the other half offer general accreditation. Most of these organizations (*n* = 19, 68%) are government-run, although four (14%) are independent entities, and five (18%) are private organizations.

The Sub-Saharan Africa region includes one of the oldest medical-education accreditors in the world, the National Universities Commission of Nigeria. This public, national agency was established in 1962 as an advisory agency and was upgraded to a statutory body in 1974. This agency has not yet applied for WFME recognition, and in fact the Sudan Medical Council (SMC) is the only WFME-recognized accreditor in this region. This leaves a large region of the world underserved by WFME-recognized medical education accreditors.

Twelve countries gained access to medical education accreditation in this region since the year 2000. However, there are seven countries in this region that only have access to general academic accreditation agencies, and 22 countries without any known form of undergraduate medical school accreditation.

### Middle East and North Africa

There are 20 countries with medical schools listed in the *World Directory* in the World Bank’s Middle East and North Africa region. We found 18 of these countries to offer some type of undergraduate accreditation, and of those, 12 utilized medical education standards. This is a 163% increase from the 11 countries with undergraduate accreditation noted by van Zanten et al. in 2008 [4].

The 18 countries with undergraduate accreditation are served by 27 separate agencies, of which 14 use medical guidelines and 13 offer only general accreditation. Many of the countries in this region have more than one accreditation agency, most notably Kuwait and Jordan, which are each served by three separate organizations. Most accreditation agencies are either run by the country’s government (*n* = 13) or are independent but government-based organizations (*n* = 12), however there are two private agencies operating in this space. One of those, The Association for Evaluation and Accreditation of Medical Education Programs (TEPDAD), is an international accreditation agency that utilizes medical guidelines and offers accreditation to three different countries in this region and four more countries in other regions of the world.

Seven countries in this region are served by an agency already recognized by WFME, and another two agencies have applications under review.

### Europe and Central Asia

This region includes 48 countries with currently operating medical schools listed in the *World Directory*, which is the largest number of countries of all World Bank regions. Similarly, this region has the highest number of accreditation organizations, with 71 agencies providing some type of undergraduate academic accreditation. Half of these agencies (*n* = 36) are independent agencies, with government organizations and private agencies each representing about a quarter of the total (*n* = 17 and *n* = 13, respectively). Accreditation coverage in this region has grown by 150% since the 2008 van Zanten et al. paper [4], and is now found in every country.

Although this region has a high number of high-income nations (58%), only 18 of these countries (38%) have medical-specific accreditation available. The remaining 62% use only general undergraduate accreditation standards.

Eleven countries in this region are covered by WFME-recognized accreditation organizations, with one additional agency’s application (in Germany) currently under review. Although Poland was the first country in this region to develop medical-specific accreditation standards, in 1997, their agency has not yet applied for WFME recognition.

### Eastern Asia and Pacific

This region includes 23 countries with medical schools listed in the *World Directory*. Of those, 21 countries have known undergraduate accreditation authorities, and more than half of those (*n* = 12) have medical-education accreditation available within their borders. Accreditation coverage in this region has increased by 233% since 2008 [4].

This region is served by 40 different undergraduate accreditation agencies, which are about equally divided between Public, Independent, and Private organizations (*n* = 11, 13, and 10, respectively), although we were unable to determine the organizational structure of six agencies. There are two countries in this region, Micronesia and North Korea, for which we were unable to find evidence of active accreditation organizations.

In this region, we see medical education accreditation forming as early as 1957, by the Philippine Accrediting Association of Schools, Colleges and Universities (PAASCU). This organization is a private, international agency, and their application for recognition by WFME is currently under review. Eight countries in this region are currently served by a WFME-recognized agency.

### Latin America and the Caribbean

There are 37 countries with active medical schools listed in the *World Directory* that are included in the World Bank’s Latin America and Caribbean region. Undergraduate accreditation systems are in place in 34 of these countries, which is a 142% increase from the number found in 2008 [4]. Of these countries, 70% (*n* = 26) have medical-education accreditation available.

The 34 countries with medical or general accreditation are serviced by 58 agencies, of which 26 are private organizations. Twenty more organizations are publicly run and funded, and we found seven independent agencies. In this region, most of the agencies (*n* = 37, 63%) use medical education-specific standards for their accreditation. Twenty countries are covered by a WFME-recognized organization, and one agency’s application for WFME recognition is currently under review, granting this region the highest rate of WFME recognition in the world.

The World Bank lists Puerto Rico as a country in the Latin American region, and this is the only nation with medical education accreditation that was formed pre-1980, through the US-based Liaison Committee on Medical Education (LCME). Three more countries (Mexico, Colombia, and Sint Maarten) gained access to medical education accreditation between 1980 and 1999. The remaining 22 countries in this region with medical education-specific accreditation have developed it in the last 20 years, making this area the 2nd largest growth region for medical education accreditation in the last two decades.

### North America

This region, with only two countries (Canada and the United States), has had medical-specific accreditation agencies in both countries since 1979. The LCME, founded in 1942, is the accrediting authority for allopathic medical education programs leading to a Doctor of Medicine (MD) degree in the US. This agency is a private organization and recognized by WFME, as is the Committee on Accreditation of Canadian Medical Schools. Also operating in this region is the Commission on Osteopathic College Accreditation (COCA), which began accreditation activities in 1952, but is not yet recognized by WFME.

## Discussion

Quality of education has been a concern since the early twentieth century and is of particular concern in medical education, as graduates of medical schools provide patient care. Medical education accreditation, first developed 60 years ago, has seen significant growth around the world in the last 20 years. While in 1980 there were only eight such accreditors, by the time of this review there were 71. High-income countries began this trend, with 11 high-income countries served by medical education accreditors before 1980. Growth has been fastest in middle-income countries, which have seen 36 countries begin using medical education accreditation in the last 20 years, but slowest in low-income countries where only six countries currently use medical education accreditation standards. Although low-income countries account for 17% (*n* = 30) of the countries in this study, they had only 6% (*n* = 7) of the medical education accreditation agencies worldwide.

These data show us that the use of medical education accreditation and standards, although increasing, is not universal. Although most countries have some type of undergraduate accreditation systems in place, the majority of these do not use standards that are specific to medical education. The Sub-Saharan African region, in particular, has a low incidence of medical education accreditation.

In countries where medical education accreditation exists, the scope and organization of the agencies varies considerably. Some international agencies were found to provide accreditation services in more than 10 countries, while others only served one or two additional countries. Latin America has a high number of private accreditation agencies, while the Middle East and North African region has only two each, and South Asia has none. In Sub-Saharan Africa, public accreditation agencies outnumber private and independent agencies by 4:1, in contrast to Europe and Central Asia where independent agencies are twice as common as either public or private organizations. This global variability in legislative formats is likely due to cultural and historical differences and is not associated with the quality or rigor of the accreditor.

Regional cooperation and international agencies seem to be a growing trend. These transnational initiatives support physician migration, mutual degree recognition, and the sharing of academic resources as technology becomes increasingly accessible. The development and recent growth of WFME recognition also indicates the spread of globalization in this area.

Our data demonstrate that medical education quality assurance systems have been increasing and improving worldwide, which should lead to evidence of more highly skilled physicians and in turn, better patient heath. For example, program data at one medical school in Saudi Arabia were analyzed following accreditation, and the authors conclude that there were significant improvements to the administration, curriculum, and educational processes [17]. In Canada, accreditation encourages medical education programs to establish processes likely to be associated with improved quality [18]. In the US, quantitative analyses have demonstrated that for certain populations of international medical graduates, graduating from an accredited medical school is associated with better performance on the United States Medical Licensing Exam (USMLE) [19, 20]. Recently, a study demonstrated that Canadian accreditation review cycles appear correlated with educational processes that are associated with better student outcomes on a national licensing examination [21]. To the extent that performance on licensing examinations is predictive of improved patient health, these studies provide evidence of a relationship between accreditation and the proficiency of graduates of accredited schools.

While this study provides an overview to medical school accreditation practices internationally, it is not without limitations. This project encountered challenges throughout the data collection and review process, such as a reliance on accreditors’ websites that may not have been updated recently or exist at all, limited access to accreditation process data for many agencies, and a pandemic that may have distracted some agencies from responding to our queries. Complex relationships and international agreements, especially those within the Central and South American region, made confirming accreditation activity for some countries difficult to discern. It is likely that agencies are operational in some countries that we did not find documentation of online. Despite our best efforts to obtain accurate data, track agencies through multiple name changes and organizational structures, and classify organizations appropriately, some agencies may have start dates considerably earlier or later than recorded, or be miscategorized, due to confusing terminology and multiple interpretations of terms such as “accreditation”, “independent”, or “autonomous”.

According to a recent review of published research on accreditation in basic medical education, accreditation agencies from high-income countries were featured most often, and most studies had at least one author from the United States or Canada [22]. As the number of accreditation agencies and their specific focus on medical education quality assurance continues to increase globally, more investigations from all regions in the world providing evidence of effectiveness are warranted. In addition, while for this study we combined medical and health profession program accreditation data, future research is needed to determine if these systems are organized and implemented the same way, within and across countries, and the impact of separation by health profession on the effectiveness of quality assurance systems.

## Conclusions

These data show us that the use of medical education accreditation and standards, although increasing, is not universal. Although most countries have some type of undergraduate accreditation systems in place, many of these do not use standards that are specific to medical education. Most accreditation systems have only developed in the last 20 years. The summary and trend data described in our study can serve as an important resource for further investigations on the effectiveness of accreditation activities worldwide, especially in areas not frequently highlighted in the literature. Descriptive data, such as type and scope of accreditation agencies and country classification statistics, can serve as a basis for frameworks for identifying and disseminating best practices. Our research also highlights regions and countries that may need focused accreditation development support.

## Data Availability

The datasets used and/or analyzed during the current study are available from the corresponding author on reasonable request.

## Appendix 1

See Table 3.

**Table 3:** Table of accreditation agencies included in this study, by country

Country	Accreditation agency
Afghanistan	Ministry of Higher Education (MoHE)
Albania	Quality Assurance Agency in Higher Education—ASCAL (Previously Public Agency for accreditation of Higher Education (PAAHE))
Algeria	National Accreditation Committee (CNH)
Angola	None
Anguilla	CAAM-HP
	ACCM
Antigua and Barbuda	CAAM-HP
	Antigua and Barbuda National Accreditation Board
Argentina	CONEAU
Armenia	ANQA
Aruba	ACCM
Australia	Australian Medical Council
Austria	AQ Austria
Azerbaijan	Education Quality Assurance Agency (Təhsildə Keyfiyyət Təminatı Agentliyinə) (MOE)
Bahrain	Bahrain Education & Training Quality Authority (BQA)
	National Authority of Qualifications and Quality Assurance for Education and Training
Bangladesh	NQAB (National Quality Assurance Body)/QAS (Quality Assurance Scheme)
Barbados	CAAM-HP
	Barbados Accreditation Council
Belarus	Education Quality Assurance Department under the Ministry of Education
Belgium (Flanders only)	The Netherlands-Flemish Accreditation Organization (NVAO)*
Belize	Ministry of Education
Benin	None
Bolivia	IAI (created by PAFAMS)
	Comisión Nacional de Evaluación y Acreditación de Carreras Universitarias (National Commission of Evaluation and Accreditation of University Professions)
Bonaire	NVAO
Bosnia and Herzegovina	Agency for Development of Higher Education and Quality Assurance
Botswana	Botswana Health Professions Council
Brazil	Accreditation System of Medicine Courses in Brazil (SAEME)
	National System of Evaluation of Higher Education (SINAES)/INEP
Bulgaria	National Evaluation and Accreditation Agency—check in EQAR/DEQAR
Burkina Faso	None
Burundi	East Africa Community (EAC)
	National Commission for Higher Education (CNES)
Cambodia	Accreditation Council of Cambodia
Cameroon	Ministry of Education University Accreditation And Quality Department (DAUQ)
Canada	Committee on Accreditation of Canadian Medical Schools (CACMS)
Cayman Islands	ACCM
Central African Republic	None
Chad	None
Chile	Comisión Nacional de Acreditación (CNA) (National Commission for Accreditation)
	Acreditadora de Chile
China	Working Committee for the Accreditation of Medical Education
Colombia	Consejo Nacional de Acreditación (National Council of Accreditation) (CNA)
Comoros	None
Congo, DRC	None
Congo, Republic	None
Cook Islands	None
Costa Rica	COMAEM
	(SINAES) Sistema Nacional de Acreditación de la Educación Superior (National System for Higher Education Accreditation)
	Institutional Evaluation Programme (IEP)
Côte d'Ivoire	None
Croatia	Agency for Science and Higher Education (AZVO)
Cuba	(SEA-CU) System of Evaluation and Accreditation of University Careers/SUPRA/(JAN) Junta de Acreditación Nacional
Curacao	ACCM
	CAAM-HP
Cyprus	The Cyprus Agency of Quality Assurance and Accreditation in Higher Education (DI.P.A.E.) (CYQAA)
Czech Republic	ASIIN
	National Accreditation Bureau for Higher Education (NAB)
Denmark	Danish Accreditation Institution
Djibouti	None
Dominica	ACCM
	Medical Board of Dominica/Ministry of Health
Dominican Republic	CAAM-HP
	MESCyT
Ecuador	(CACES) Consejo de Evaluación, Acreditación y Aseguramiento de la Calidad de la Educación Superior (Board of Assessment, Accreditation and Quality Assurance in Higher Education) (CEAACES)/Consejo de Aseguramiento de la Calidad de la Educación Superio (CACES)
Egypt	National Authority for Quality Assurance and Accreditation of Education (NAQAAE)
El Salvador	Comisión de Acreditación de la Calidad de la Educación Superior (Commission of Accreditation of the Quality of Higher Education)
Eritrea	None
Estonia	Estonian Quality Agency for Higher and Vocational Education (EKKA)
Ethiopia	Higher Education Relevance and Quality Agency (HERQA)
Fiji	FHEC
	Educational Quality and Assessment Programme/Secretariat of the Pacific Community (EQAP)
Finland	Finnish Education Evaluation Center (FINEEC)
France	HCERES—High Council for Evaluation of Research and Higher Education
Gabon	None
Gambia	National Accreditation and Quality Assurance Authority (NAQAA)
Georgia	National Center for Educational Quality Enhancement (NCEQE)
	ASIIN
Germany	AAQ (Swiss)
	ASIIN
	Stiftung Akkreditierungsrat
	AQAS
	ACQUIN
Ghana	National Accreditation Board
Greece	Hellenic Quality Assurance and Accreditation Agency
Grenada	CAAM-HP
	Grenada Medical and Dental Council
	Grenada National Accreditation Board
Guadeloupe	None
Guatemala	None
Guinea	None
Guinea Bissau	None
Guyana	CAAM-HP
	National Accreditation Council
Haiti	None
Honduras	Consejo de Educacion Superior
Hong Kong	Medical Council of Hong Kong/Education and Accreditation Committee
Hungary	Hungarian Accreditation Committee
Iceland	Ministry of Education, Science and Culture
	Quality Board for Icelandic Higher Education
India	National Assessment and Accreditation Council
Indonesia	Indonesian Accreditation Agency for Higher Education in Health (IAAHEH/LAM—PTKes)
	ASIIN
	ASEAN University Network—QA (AUN-QA)
	National Accreditation Agency for Higher Education (BAN-PT)
Iran	Secretariat of the Council for Undergraduate Medical Education (part of the Ministry of Health and Medical Education)
Iraq	National Council for Accreditation of Medical Colleges (NCAMC)/Apparatus of Supervision and Scientific Evaluation (ASSE)
Ireland	Irish Medical Council
Israel	Council for Higher Education
Italy	National Agency for the Evaluation of Universities and Research Institutes
Jamaica	CAAM-HP
Japan	Japan Accreditation Council for Medical Education (JACME)
	Japan University Accreditation Association
	Japan Institution for Higher Education Evaluation
Jordan	ACCM
	Accreditation and Quality Assurance Commission for Higher Education Institution (AQACHEI)
	Higher Education Accreditation Commission
Kazakhstan	Independent Agency for Accreditation and Rating (IAAR)
	Eurasian Centre for Accreditation and Quality Assurance in Higher Education and Health Care (ECAQA)
	The Association for Evaluation and Accreditation of Medical Education Programs (TEPDAD) Tıp Eğitimi Değerlendirme ve Akreditasyon Derneği
	Independent Kazakhstan Quality Assurance Agency in Education (IQAA)
Kenya	East Africa Community (EAC)
	Commission for University Education
Kosovo	Kosovo Accreditation Agency (KAA)
Kuwait	The Association for Evaluation and Accreditation of Medical Education Programs (TEPDAD) Tıp Eğitimi Değerlendirme ve Akreditasyon Derneği
	National Bureau for Academic Accreditation & Education Quality Assurance (NBAQ)
	Private Universities Council (PUC)
Kyrgyzstan	IAAR
	National Accreditation Council
	Independent Agency for the Accreditation of Educational Programs and Organizations (AAEPO)
	Eurasian Centre for Accreditation and Quality Assurance in Higher Education and Health Care (ECAQA)
	Agency for Quality Assurance in Education "EdNet"
	Independent Accreditation Agency "Bilim—Standart" (n.d.); Independent Accreditation Agency "El Baasy" (n.d)
Laos (Lao People’s Democratic Republic)	ESQAC (Education Standards and Quality Assurance Center)/CEQA (Center for Quality Assurance)
	AUN-QA
Latvia	Quality Agency for Higher Education of the Academic Information Center
Lebanon	The Association for Evaluation and Accreditation of Medical Education Programs (TEPDAD) Tıp Eğitimi Değerlendirme ve Akreditasyon Derneği
	MoHE
Liberia	National Commission on Higher Education
Libya	National Center for Quality Assurance and Accreditation of Educational and Training Institutions (NCQAA)
Lithuania	Centre for Quality Assessment in Higher Education (SKVC)
Macau, S.A.R. China	Higher Education Bureau Tertiary Education Services Office
Madagascar	None
Malawi	National Council of Higher Education
Malaysia	Malaysian Qualifications Agency
	ASEAN University Network—QA (AUN-QA)
Maldives	Maldives Medical and Dental Council
	Maldives Qualifications Authority
Mali	None
Malta	ASIIN
	National Commission for Further and Higher Education (NCFHE)
Mauritania	None
Mauritius	Tertiary Education Commission
Mexico	Consejo Mexicano para la Acreditación de la Educación Médica (COMAEM) (Mexican Board for Accreditation of Medical Education)
	ASIIN
	IAI
Micronesia	None
Moldova	IAAR
Mongolia	ASIIN
	The National Council for Education Accreditation
Montenegro	Agency for Quality Control and Quality Assurance in Higher Education
Montserrat	CAAM-HP
Morocco	National Agency of Assessment and Quality Assurance in Higher Education and Scientific Research (ANEAQ)
Mozambique	National Council on Quality Assurance in Higher Education (CNAQ)
Myanmar	Myanmar Medical Council
	National Accreditation & Quality Assurance Committee (NAQAC) Department of Higher Education, Ministry of Education
Namibia	Health Professions Council of Namibia Education and Training Quality Assurance Department
	Namibia Qualifications Agency
Nepal	Nepal Medical Council
Netherlands	The Netherlands-Flemish Accreditation Organization (NVAO)
New Zealand	Medical Council of New Zealand (together with the Australian Medical Council)
Nicaragua	Consejo Nacioncal de Evaluacion y Acreditacion (CNEA)
Niger	None
Nigeria	National Universities Commission
North Korea	None
North Macedonia	Board for Accreditation and Evaluation
Norway	Norwegian Agency for Quality Assurance in Education (NOKUT)
Oman	The Association for Evaluation and Accreditation of Medical Education Programs (TEPDAD) Tıp Eğitimi Değerlendirme ve Akreditasyon Derneği
	Oman Academic Accreditation Authority (OAAA)
Pakistan	Pakistan Medical and Dental Council
	Higher Education Commission
Palestinian Authority	The Association for Evaluation and Accreditation of Medical Education Programs (TEPDAD) Tıp Eğitimi Değerlendirme ve Akreditasyon Derneği
	Accreditation and Quality Assurance Commission (AQAC)
Panama	(CONEAUPA) National Council for the Evaluation and Accreditation of University Education in Panama (Consejo Nacional de Evaluación y Acreditación Universitaria de Panamá)
	Instituto Acreditación Internacional (IAI)
Papua New Guinea	Department of Higher Education, Research, Science and Technology (DHERST)
Paraguay	Agencia Nacional de Evaluación y Acreditación de la Educación Superior (National Agency of Evaluation and Accreditation of Higher Education) (ANEAES)
Peru	(SINEACE) National System for Evaluation, Accreditation and Certification of Educational Quality
Philippines	Philippine Accrediting Association of Schools, Colleges and Universities (PAASCU)
	ASEAN University Network—QA (AUN-QA)
	Commission on Higher Education
Poland	University Commission for the Quality of Medical Education (UKJKKL)
	Polish Accreditation Committee (PKA)
Portugal	Agency for Assessment and Accreditation of Higher Education (A3ES)
Puerto Rico	LCME
Qatar	The Association for Evaluation and Accreditation of Medical Education Programs (TEPDAD) Tıp Eğitimi Değerlendirme ve Akreditasyon Derneği
Romania	ASIIN
	Romanian Agency for Quality Assurance in Higher Education (ARACIS)
Russia	Independent Agency for Accreditation and Rating (IAAR)
	ASIIN
	National Accreditation Agency (NAA)
	National Centre for Public Accreditation (NCPA)
	Agency for Quality Assurance in Higher Education and Career Development (AKKORK)
Rwanda	Rwanda Medical Dental Council
	Higher Education Council
Saba	The Netherlands-Flemish Accreditation Organization (NVAO)
Saint Kitts and Nevis	ACCM
	CAAM-HP
Saint Lucia	CAAM-HP
Saint Vincent and the Grenadines	ACCM
	CAAM-HP
Samoa	Philippine Accrediting Association of Schools, Colleges and Universities (PAASCU)
Saudi Arabia	National Commission for Academic Accreditation and Assessment (NCAAA)
	Ministry of Education—Higher Education
Senegal	None
Serbia	ASIIN
	(NAT) National Body for Accreditation and Quality Assurance in Higher Education/(KAPK) Commission for Accreditation and Quality Assurance
Sierra Leone	None
Singapore	ASEAN University Network—QA (AUN-QA)
Sint Maarten	Accreditation Commission on Colleges of Medicine (ACCM)
Slovakia	(SAAVS) Slovak Accreditation Agency for Higher Education/Accreditation Commission
Slovenia	Slovenian Quality Assurance Agency for Higher Education
Somalia	None
South Africa	HPCSA
	Higher Education Quality Committee/Council of Higher Education
South Korea	Korean Institute of Medical Education and Evaluation (KIMEE)
South Sudan	East Africa Community (EAC)
Spain	Agencia Nacional de Evaluación de la Calidad y Acreditación (ANECA) (National Agency for Quality Assessment and Accreditation)
Sri Lanka	Quality Assurance and Accreditation Council of the University Grants Commission
Sudan	Sudan Medical Council (SMC)
	Accreditation Committee for Accreditation of Medical Schools (formed jointly by the Higher Education Ministry Committee and the Sudan Medical Council)
Suriname	Nationaal Orgaan voor Accreditatie (NOVA)
Sweden	Swedish Higher Education Authority
Switzerland	Swiss Agency for Accreditation and Quality Assurance (AAQ) (Swiss Accreditation Council of the FDHA)
Syria	Ministry of Higher Education
Taiwan	Taiwan Medical Accreditation Council
Tajikistan	IAAR
Tanzania	East Africa Community (EAC)
	Tanzanian Commission for Universities
Thailand	Institute for Medical Education Accreditation (IMEAC)
	ASEAN University Network—QA (AUN-QA)
	Office of the Higher Education Commission
Togo	None
Trinidad and Tobago	CAAM-HP
	Accreditation Council of Trinidad and Tobago (ACTT)
Tunisia	National Evaluation, Quality Assurance and Accreditation Authority
Turkey	The Association for Evaluation and Accreditation of Medical Education Programs (TEPDAD) Tıp Eğitimi Değerlendirme ve Akreditasyon Derneği
Turkmenistan	Ministry of Education
Uganda	East Africa Community (EAC)
	Uganda National Council for Higher Education
Ukraine	Ministry of Education and Science of Ukraine
	National Agency for Higher Education Quality Assurance
United Arab Emirates	Commission for Academic Accreditation
United Kingdom	GMC Education Committee
United States	Commission on Osteopathic College Accreditation (COCA), American Osteopathic Association
	Liaison Committee on Medical Education (LCME)
Uruguay	ARCU-SUR
Uzbekistan	State Testing Center
Venezuela	None
Viet Nam	Ministry of Education and Training Department of Education Testing and Accreditation
	Center for Education Accreditation, Viet Nam National University Ha Noi (VNU-CEA)
	Centre for Education Accreditation of the Association of Vietnam Universities and Colleges (CEA-AVU&C)
	IIG Viet Nam
	International Council for Higher Education Accreditation (ICHEA)
	ASEAN University Network—QA (AUN-QA)
Yemen	None
Zambia	Health Professions Council of Zambia
Zimbabwe	None

Information was accurate and confirmed as of August 1, 2020

## Abbreviations

COCA: Commission on Osteopathic College Accreditation
DORA: Directory of Organizations that Recognize/Accredit medical schools
DEQAR: Database of External Quality Assurance Results
EQAR: European Quality Assurance Register
FAIMER: Foundation for Advancement of International Medical Education and Research
INQAAHE: International Network for Quality Assurance Agencies in Higher Education
LCME: Liaison Committee on Medical Education
PAASCU: Philippine Accrediting Association of Schools, Colleges and Universities
SMC: Sudan Medical Council
TEPDAD: The Association for Evaluation and Accreditation of Medical Education Programs
USMLE: United States Medical Licensing Exam
World Directory: World Directory of Medical Schools
WFME: World Federation for Medical Education
